# Sexual and gender harassment and use of psychotropic medication among Swedish workers: a prospective cohort study

**DOI:** 10.1136/oemed-2021-108087

**Published:** 2022-03-09

**Authors:** Katrina Julia Blindow, Johan Paulin, Linda Magnusson Hanson, Kristina Johnell, Anna Nyberg

**Affiliations:** 1 Institute of Environmental Medicine, Karolinska Institutet, Stockholm, Sweden; 2 Department of public health and caring sciences, Uppsala University, Uppsala, Sweden; 3 Department of Psychology, Stockholms Universitet, Stockholm, Sweden; 4 Department of Medical Epidemiology and Biostatistics, Karolinska Institutet, Stockholm, Sweden

**Keywords:** mental health, occupational health, violence, men, women

## Abstract

**Objective:**

To estimate the prospective association between the exposure to three types of gender-based violence and harassment (GBVH) and psychotropic medication.

**Methods:**

Information on three measures of workplace GBVH—sexual harassment (1) from superiors or colleagues, (2) from others (eg, clients) and (3) gender harassment from superiors or colleagues—were retrieved from the biannual Swedish Work Environment Survey 2007–2013 (N=23 449), a representative sample of working 16–64 years old registered in Sweden. The survey answers were merged with data on antidepressants, hypnotics/sedatives and anxiolytics from the Swedish Prescribed Drug Register. Cox proportional hazards analyses with days to purchase as time scale and first instance of medicine purchase as failure event were fitted, adjusted for demographic and workplace factors.

**Results:**

Workers who reported exposure to gender harassment only (HR 1.2, 95% CI 1.07 to 1.36), to sexual but not gender harassment (HR 1.21, 95% CI 1.04 to 1.40), or to gender and sexual harassment (HR 1.31, 95% CI 1.08 to 1.60) had an excess risk of psychotropics use in comparison to workers who reported neither of the exposures in the past 12 months. We found no interaction between the exposures and gender in the association with psychotropics use.

**Conclusions:**

Exposure to sexual or gender harassment at the workplace may contribute to the development of mental disorders.

**Key messages**

**What is already known about this subject?**

- There is some evidence that workplace exposure to sexual harassment is prospectively associated with self-reported mental ill health. Less attention has been paid to the effect of gender harassment—work-related experiences of non-sexualising sexism—on mental health. Neither sexual nor gender harassment have previously been investigated as risk factors for the use of psychotropic medication.

**What are the new findings?**

- Swedish workers who are exposed to work-related sexual or gender harassment have an excess risk of psychotropic medication use.

**How might this impact on policy or clinical practice in the foreseeable future?**

- Organisations need to pay more attention not only to sexual but also gender harassment in the workplace, and health care professionals would be well advised to consider sexual and gender harassment as potential factors in the aetiology of common mental disorders.

## Introduction

Mental ill health is a major problem worldwide, depression was first and anxiety sixth on the WHO global ranking on contributors to disability in 2015.^1^ In the Swedish workforce, this is reflected in a continuous rise in sickness absence due to psychiatric diagnoses in the past three decades, now the most common diagnoses leading to long-term sickness absence.^2^ This trend is driven mostly by depression, anxiety and stress-related mental disorders.^2^ In addition to the immediate suffering, episodes of mental ill health can seriously disrupt for example, a person’s labour market attachment,^3^ life-time earnings^4^ or family situation.^5^

Experiences with adverse social behaviour at work, such as violence, harassment or bullying can cause the affected considerable distress.^6 7^ It can be difficult for victims to distinguish the grounds for the mistreatment. When they attribute the mistreatment to their gender, this constitutes a case of gender-based violence and harassment (GBVH) as defined by the International Labor Organization (ILO).^8^ Sexual harassment is highlighted by the ILO as a specific kind of GBVH and has been recognised by occupational health research as a potentially harmful workplace exposure for several decades.^9–11^ Non-sexualising forms of GBVH, also included in the ILO definition of ‘harassment and violence directed at persons because of their sex or gender’, on the other hand have only recently gained attention under the construct of gender harassment.^12–15^ While sexual harassment is often experienced in combination with non-sexualising expressions of sexist hostility, gender harassment is far more common in workplaces and occurs often in the absence of sexualising offences.^12 14–16^ In this study, we investigate (1) self-labelled sexual harassment, defined as unwanted advances and offensive remarks of a sexual nature and (2) experiences of gender harassment, defined as non-sexualising sexist offences and expressions of disrespect that the affected perceived as based on their gender.^12^

Differences in definitions, measurement and under-reporting complicate determining the prevalence of work-related GBVH.^9 17 18^ In the Swedish Work Environment Reports from 1999 to 2013 at least one experience of sexual or gender harassment in the past 12 months was reported by about 18% of women and 6% of men.^19^ Associations of work-related GBVH based on diverse measures of sexual harassment, gender harassment or related constructs with diminished mental health have been found repeatedly.^11 15 20 21^ Most studies, however, suffer methodological limitations, as they are cross-sectional and measure exposure and outcome with self-reports.^9 15 22^ To the best of our knowledge, all prospective studies that have investigated the association of sexual harassment with self-reported mental health outcomes found associations in women, while in men, the results have been less consistent.^23–25^ In the only prospective study using register data on mental ill health (based on the same survey items for sexual harassment as this study), an increased risk of suicide attempts and suicides in victims of work-related sexual harassment was found.^26^ To our knowledge, the prospective relationship between work-related gender harassment and mental ill health has not previously been investigated, and no study so far has explored the prospective association of sexual or gender harassment with the use of psychotropic medication.

The frequency with which the harassment is experienced has rarely been investigated as a key predictor of the impact it has on the mental health of the affected.^7 15^ As to fill this gap, we distinguish reports of one-time experiences from reoccurring exposure in this study. Also, in some studies, the association of sexual harassment and mental ill health was found to differ depending on whether the victim was harassed by workplace personnel or others.^27 28^ Therefore, we distinguish between sexual harassment by (1), superiors or colleagues and (2), others (eg, clients) where possible. Finally, we investigate gender harassment from superiors or colleagues as a risk factor for psychotropics use.

## Methods

### Design and study population

For this prospective cohort study, we pooled cross-sectional survey data for exposure measurement and connected follow-up register data on the purchase of psychotropic medication. The study is based on participants of the Swedish Work Environment Survey (SWES) 2007–2013 (N=26 327). SWES is a biannual cross-sectional survey containing questions about respondents’ work environment, conducted by Statistics Sweden. SWES participants are a random subsample from the Labour Force Survey (LFS), which is conducted by telephone with about 20 000 individuals, randomly selected from the Swedish population after stratification for gender, country of birth and employment status. Individuals eligible for participation in SWES fulfilled the additional criteria that they were 16–64 years of age, in paid work (≥1 hours/week) and not on long-term sick leave or absent from work for other reasons in the 3 months prior to data collection. They received a paper survey by mail. The response rate decreased from 71% in 2007 to 58% in 2013.^29^ SWES survey participants largely represented the general population with regards to gender, age, education and income distribution, while individuals born outside Sweden are underrepresented.^30^ To capture incident medication after reporting GBVH, we excluded 2875 individuals who had purchased psychotropic medication in the calendar year of survey participation. This gave 23 452 participants, of whom 23 449 individuals had complete data on their registered gender and the purchase of medication. Among those, 1.3% had missing values for gender harassment, 0.7% for sexual harassment from superiors or colleagues, and 0.9% for sexual harassment from others (eg, clients). Missing values in the exposure variables were not associated with psychotropic medication. In the different analyses, between 1% and 3.8% of the participants were additionally excluded due to missing values in study variables.

### Study variables

#### Filling of prescription for psychotropic medication

Information on the psychotropic medication, including date of dispense and type of medication, was retrieved from the Swedish Prescribed Drug Register. The register is maintained by the Swedish National Board of Health and Welfare and contains data on prescribed medication dispensed at pharmacies in Sweden. Based on the Anatomical Therapeutic Chemical classification system prescriptions for antidepressant (N06A), anxiolytics (N05B) and hypnotics/sedatives (N05C) were included and subsumed in one variable for psychotropic medication. We did not include antipsychotics (N05A), as they are less commonly prescribed for the treatment of stress-related diagnoses. Data were available 1 July 2005–31 January 2017. Depending on the year of survey participation, this provided a follow-up time of at least 3 years and 1 month, at most 9 years and 1 month and a mean follow-up time of 6 years and 4.5 months.

#### Workplace sexual and gender harassment

The survey provided the following definition for sexual harassment: ‘Sexual harassment refers to unwanted advances or offensive remarks generally associated with sex’. This was followed by two questions: ‘Are you subjected to sexual harassment in your workplace from… (1) superiors or colleagues? and (2) other people (eg, patients, clients, passengers, students)?’. We considered the first item as sexual harassment from superiors or colleagues and the second as sexual harassment from others (eg, clients). The item for gender harassment from superiors or colleagues followed directly with the description: ‘The next question concerns whether you have experienced conduct (other than that described above) which is based on your gender and that hurts your integrity or is degrading. This can be for example, condescending and ridiculing remarks about men or women in general or in the context of your profession. It can also mean that somebody does not take notice of you or of your contributions because of your gender.’ The question read: ‘Are you subjected to harassment based on your gender in your workplace from superiors or colleagues?’. The three items were rated individually on a seven-point Likert-type scale ranging from ‘Not at all in the last 12 months’ to ‘Every day’. Based on dichotomous exposure variables (‘Not at all in the last 12 months’ indicating no exposure and any exposure frequency indicating exposure), we combined the three exposure variables into one variable for GBVH with four categories: (1) No exposure; (2) Exposure to gender harassment but not sexual harassment, (3) Sexual harassment but not gender harassment and (4) Exposure to sexual and gender harassment. As to investigate the role of exposure frequency, the two variables for sexual harassment and the variable for gender harassment were categorised into three groups: ‘Not in 12 months’, ‘Once in 12 months’ and the five following categories grouped as ‘Monthly to daily’. In addition, we generated a compound variable for Workplace sexual harassment, combining the two sexual harassment exposures. The three categories were kept and the highest value in either sexual harassment variable determined the value in the compound variable.

#### Covariates

Information on managerial responsibilities was gained from the telephone interview (LFS), where respondents were asked if they had managerial responsibilities, with response options ‘yes’ and ‘no’. All other information was retrieved from the Swedish Longitudinal Integrated Database for Health Insurance and Labour Market Studies (LISA). LISA contains data on all individuals who are registered in Sweden and are 16 years or older and can be connected to SWES through the Swedish personal identification number.^31^ Gender was categorised as woman or man in accordance with the registered gender in the year of survey participation. Age was grouped into five categories: 16–25, 26–35, 36–45, 46–55, 56–64 years. Parental migration background was dichotomised into ‘One or both parents born in Sweden’ and ‘Both parents born outside of Sweden’. Education was listed as: ‘compulsory’, ‘2-year upper secondary’, ‘3–4 years upper secondary’, ‘university <3 years’ and ‘university ≥3 years’. Disposable income was categorised into quartiles. Family situation was grouped as ‘single/divorced/widowed, no children’, ‘single/divorced/widowed with children’, ‘married/living with partner, no children’ and ‘married/living with partner with children’. We also used a variable with seven categories for *industry classification* by main activities and gender composition in accordance with Cerdas *et al*.^29^ Finally, we included a variable for the year of survey participation (2007, 2009, 2011 or 2013).

### Analytical strategy

#### Main analysis

To estimate the association between the respective GBVH exposures and the incident use of psychotropic medication, we performed Cox proportional hazards analyses with days to filled prescription as the time-scale and first instance when a psychotropic was dispensed as the failure event. The proportional hazards assumption was tested with Kaplan-Meier plots and based on Schoenfeld residuals, with no deviations from proportionality. All analyses were adjusted for survey year, gender, age, education, family situation, income, parental migration background, managerial responsibilities and industry classification. We performed the Cox regression analyses (1) for the compound measure with different combinations of sexual and gender harassment (GBVH) and (2) separately for the four independent variables using ‘Not in 12 months’ of the respective exposure variable as the reference.

#### Additional analysis

To estimate indications of a dose–response relationship between exposure and outcome, we performed analyses in which the respective exposure variable was treated as continuous. A statistically significant coefficient was interpreted as an indication of a dose–response relationship. Furthermore, we tested whether the associations were modified by gender or age by stratified analyses and by introducing interaction terms into the main models. Finally, we performed two sensitivity analyses. First, we fitted the main models with the follow-up time censored to 2 years. Second, we conducted the analyses with the full study sample, not excluding individuals who had purchased medication in the survey year and adjusting instead for previous psychotropics use. All analyses were performed using Stata V.16.1 (StataCorp).

## Results

Descriptive statistics are presented in table 1. Gender harassment was reported by 1337 (11.2%) women and 478 (4.2%) men, sexual harassment from superiors or colleagues by 249 (2.1%) women and 104 (0.9%) men, and sexual harassment from others (eg, clients) by 797 (6.7%) women and 190 (1.7%) men. In total, of those 353 workers experiencing sexual harassment from superiors or colleagues, 32% also reported sexual harassment from others and 63.7% gender harassment from superiors or colleagues. Among the 987 workers who reported sexual harassment from others (eg, clients), 29.4% also reported gender harassment from superiors or colleagues.

**Table 1:** Distribution of study variables in the first column, percentages of those dispensed psychotropic medication under follow-up in second and prevalence of workplace sexual harassment (SH) and gender harassment (GH) in third and fourth column

	All	Psychotropics	GH	SH
	N (%)	%	%	%
**All**	23 452 (100)	18.5	7.5	5.2
**Psychotropics**	4 333 (18.5)			
Anxiolytics	1 297 (29.9-)	–	9.5	6.3
Hypnotics/sedatives	1 632 (37.7)	–	8.7	6.5
Antidepressants	1 404 (23.4)	–	11.3	8.6
No medication	19 119 (81.5)	–	7.3	4.8
**Gender**				
Women	11 929 (50.9)	22.8	11.2	8.1
Men	11 520 (49.1)	14	4.2	2.2
Missing	3	–	–	–
**Age (years**)				
16–25	1 957 (8.3)	16.4	9.1	11.7
26–35	4 183 (17.8)	17.9	11.0	8.3
36–45	6 077 (25.9)	18.4	8.3	4.5
46–55	6 214 (26.5)	18.9	6.6	4.2
56–64	4 986 (21.3)	19.4	5.4	2.2
Missing	35 (0.2)	14.3	2.9	–
**Parental migration background**				
One or both parents born in Sweden	20 966 (89.4)	18.1	7.5	5.0
Parents born outside Sweden	2 485 (10.6)	21.3	9.6	6.7
Missing	1 (0.0)	–	–	–
**Family situation**				
Single/divorced/widowed, no children	6 441 (27.5)	18.1	9.4	7.3
Single/divorced/widowed with children	1 750 (7.5)	24.1	11.8	7.5
Married/living with partner, no children	4 204 (17.9)	19.7	5.6	2.7
Married/living with partner with children	11 054 (47.1)	17.3	7.0	4.6
Missing	4	–	–	–
**Education**				
Compulsory	3 006 (12.8)	22.4	4.7	4.3
2-year upper secondary	4 936 (21.1)	17.7	4.8	3.7
3–4 years upper secondary	6 771 (28.9)	17.0	8.0	6.3
University <3 years	3 207 (13.7)	18.4	10.0	5.7
University ≥3 years	5 522 (23.6)	18.9	10.5	5.4
Missing	10 (0.0)	20.0	–	–
**Managerial responsibilities**				
Yes	7 067 (30.1)	17.2	8.2	4.7
No	16 193 (69.1)	19.1	7.5	5.4
Missing	70 (23.2)	15.1	9.9	4.7
**Industry classification**				
Education	3 020 (12.9)	20.3	8.2	3.8
Health and social care	3 949 (16.8)	24.4	8.1	11.7
Labour intensive services	5 201 (22.2)	19.4	8.1	6.4
Knowledge intensive services	3 019 (12.9)	16.5	7.7	2.5
Public administration	1 652 (7.0)	17.6	10.5	4.8
Goods and energy production	3 507 (15.0)	15.0	7.3	1.7
Machine operations	2 759 (11.8)	13.1	5.5	3.0
Missing	345 (1.5)	20.9	4.9	5.5
**Disposable income (SEK**)				
≤SEK176 499	4 411 (18.8)	21.8	7.4	8.4
SEK176 500–SEK226 199	5 333 (22.7)	21.0	8.6	6.7
SEK226 200–SEK287 099	6 544 (27.9)	17.7	8.3	4.9
≥2 87 100	7 161 (30.5)	15.2	6.9	2.4
Missing	3 (0.0)	–	–	–
**Survey year**				
2007	6 841 (29.2)	24.4	7.9	5.6
2009	5 593 (23.9)	19.8	7.9	5.7
2011	6 894 (29.4)	16.0	7.3	4.7
2013	4 124 (17.6)	11.1	8.1	4.8
Missing	–	–	–	–

### Sexual and gender harassment and risk of psychotropic medication

#### Combinations of sexual and gender harassment

As presented in table 2, we found an excess risk of psychotropics use in workers who reported exposure to gender harassment only (HR 1.2, 95% CI 1.07 to 1.36), sexual but not gender harassment (HR 1.21, 95% CI 1.04 to 1.40), and to gender and sexual harassment (HR 1.31, 95% CI 1.08 to 1.60) in comparison to workers who had been exposed to neither kind of GBVH in the past 12 months.

**Table 2:** Results from Cox regression analyses on the association between different combinations of work-related sexual and gender harassment (GBVH) and psychotropics, presented as HRs and 95% CIs

	Exposed	Psychotropics	
	N (%)	N	HR (95% CI)
**GBVH**			
Not in 12 months	19 928 (88.7)	3 537	1
Gender harassment/no sexual harassment	1352 (6.0)	307	1.20 (1.07 to 1.36)
Sexual harassment/no gender harassment	769 (3.4)	193	1.21 (1.04 to 1.40)
Sexual and gender harassment	418 (1.9)	106	1.31 (1.08 to 1.60)

Adjusted for survey year, gender, age, education, family situation, income, parental migration background, managerial responsibilities and industry classification.

GBVH, gender-based violence and harassment.

#### One-time and reoccurring harassment experiences

The results for the associations of the three exposure measures for work-related sexual harassment, gender harassment and the incident use of psychotropics are presented in table 3. Exposure monthly to daily was associated with psychotropics use in the composite measure of workplace sexual harassment (HR 1.37, 95% CI 1.12 to 1.67), sexual harassment from superiors or colleagues (HR 1.40, 95% CI 1.00 to 1.96) and sexual harassment from others (eg, clients) (HR 1.39, 95% CI 1.12 to 1.73). Compared with respondents who had not experienced gender harassment in the past 12 months, an elevated risk of psychotropics use was found in those who experienced gender harassment once in 12 months (HR 1.23, 95% CI 1.09 to 1.39). When the respective exposure variables were treated as continuous, a statistically significant coefficient was found in all four exposure variables, suggesting a linear association with psychotropic medication.

**Table 3:** Results from Cox regression analyses on the association between work-related sexual harassment (SH) and gender harassment and psychotropics, presented as HRs and 95% CIs

	Exposed	Psychotropics	
	N (%)	N	HR (95% CI)
**Workplace SH by any harasser**			
Not in 12 months	21 280 (94.7)	3844	1
Once in 12 months	798 (3.6)	196	1.15 (0.99 to 1.33)
Monthly to daily	389 (1.7)	103	1.37 (1.12 to 1.67)
**SH by superiors or colleagues**			
Not in 12 months	22 125 (98.5)	4 061	1
Once in 12 months	207 (0.9)	48	1.13 (0.85 to 1.51)
Monthly to daily	135 (0.6)	34	1.40 (1.00 to 1.96)
**SH by others (eg, clients**)			
Not in 12 months	21 512 (95.8)	3 898	1
Once in 12 months	658 (2.9)	163	1.14 (0.98 to 1.34)
Monthly to daily	297 (1.3)	82	1.39 (1.12 to 1.73)
**Gender harassment**			
Not in 12 months	20 697 (92.1)	3730	1
Once in 12 months	1 205 (5.4)	284	1.23 (1.09 to 1.39)
Monthly to daily	565 (2.5)	129	1.17 (0.98 to 1.40)

Adjusted for survey year, gender, age, education, family situation, income, parental migration background, managerial responsibilities and industry classification.

#### Effect-modification by gender and age

We found no interaction of sexual harassment or gender harassment with gender in the association with psychotropics use, and no interaction of sexual harassment with age (for gender-stratified and age-stratified results see online supplemental material 1). For gender harassment, the age-stratified analysis gave similar results in all age groups, except in the youngest (16–25), where no association between gender harassment once in 12 months and psychotropics was found (HR 0.70, 95% CI 0.42 to 1.17). Interaction analysis confirmed a stronger association between gender harassment once in 12 months and psychotropics in 26–35 years old (HR 1.89, 95% CI 1.08 to 3.31) and 36–45 years (HR 1.72, 95% CI 1.00 to 2.96) compared with young workers (age 16–25).

10.1136/oemed-2021-108087.supp1 Supplementary data

#### Sensitivity analyses

Analyses censored to 2-year follow-up gave similar effect sizes as the analyses with full follow-up, but with lower precision. Alternative analyses adjusting for previous psychotropics use (instead of the exclusion of individuals with psychotropics in the survey year) resulted in similar effect sizes with higher precision (for results from sensitivity analyses, see online supplemental material 1).

## Discussion

### Main findings

We followed a sample of the Swedish working population for on average 6 years and 4.5 months and found an excess risk of psychotropics use in workers who had experienced sexual or gender harassment.

### Comparison to prior studies and interpretation

To our knowledge, this is the first study that examined the prospective association between sexual and gender harassment and psychotropic medication. The purchase of psychotropic medication can be interpreted as a measure of mental health disorders of a certain severity. The relationship between mental ill health and the use of psychotropic medication is however far from straightforward. A study in the Stockholm region found 47% of participants who fulfilled the diagnostic criteria for depression or anxiety disorder had been in contact with healthcare for psychological symptoms in the past year. The Swedish National Board of Health and Welfare estimates that approximately 50% of patients with a diagnosis of a depressive or anxiety disorder are prescribed antidepressants, and that approximately 66% of those collect the prescribed medication.^32^

#### Sexual and gender harassment and the risk of psychotropics use

Four previous studies investigated the prospective association between sexual harassment and mental health outcomes.^23–25 28^ Three of these did not specify if the harasser was a co-worker or someone else (eg, a client).^23–25^ Their results relate most closely to our compound measure of workplace sexual harassment. All three studies found pronounced associations of sexual harassment with prospective mental ill health. Our results confirm these previous findings and suggest that the effect is so severe and long-lasting that it gives grounds for treatment. Rugulies *et al* 28 conducted analyses distinguishing harassment from workplace personnel (eg, coworkers) and others (eg, clients). They found elevated depressive symptoms in respondents who experienced sexual harassment from workplace personnel and those harassed by others, but with depressive disorder only in respondents exposed by workplace personnel. In this study, the HRs for harassment from superiors or colleagues and from others (eg, clients) were very similar. However, statistical power was limited in some analyses, and the results are inconclusive.

Research specifically on gender harassment is still scarce. A recent meta-analysis suggests that sexual harassment and gender harassment are equally harmful, and that pervasiveness of the experience is crucial.^15^ Our results partly confirm this assessment. The results for sexual and gender harassment were very similar. The role of pervasiveness of the experience was not clear, though. While reoccurring exposure to sexual harassment was more strongly related to psychotropics use than one-time exposure, the opposite was true for gender harassment. This points to a difficulty with the survey items. While exposure frequency is a good measure of pervasiveness, we have no information on severity. Particularly the definition of gender harassment provided in the survey encompasses a spectrum from blatantly (hetero)sexist to more covert and ambiguous conduct. Some respondents might have recalled incidents that only occurred once but had severe consequences, while some reported reoccurring incidences that they experienced as inconsequential.

#### Gender and age

Previous literature indicates considerable differences between the constellations where the harassment of women and men occurs.^30 33^ Women and men were also found to differ in the types of conduct they predominantly experience, and the experiences they consider offensive and tend to self-label as sexual harassment.^11 17 33–36^ At the same time, men have been found to recognise depression to a lesser extent than women, regardless if they showed symptoms,^37^ perceive less need for mental health care (after adjustment to mental well-being) and to be less inclined to seek care than women.^38^ They might also receive treatment with psychotropic medication less often when presenting with similar diagnoses as women.^39^

Previous studies that investigated gender differences all found an association in women, but the findings were less consistent in men. Sterud and Hanvold^25^ found a stronger association of sexual harassment with mental distress in men than in women. In contrast, Nielsen and Einarsen^24^ found no prospective association with distress, but mental distress predicted reports of sexual harassment in men. Houle *et al* 23 found no indication of gender differences. We found no indications of gender differences in the association of sexual or gender harassment with psychotropics use. Due to fewer harassment cases in combination with almost half the incidences in our outcome measure in men compared with women, our possibility to find a true association in men and to investigate gender-differences was limited, though. Future research needs to take the gendered character of health symptoms and health behaviour into account and investigate typically male outcomes, for example, substance abuse and accidents.

Though young workers are consistently found to be disproportionally exposed to sexual harassment,^6^ the role of age for how the mistreatment affects workers has gained little attention.^40^ We found no interaction of age with sexual harassment, but with gender harassment. We can only speculate, why we found no association in the youngest workers. Many young people might still focus on their education and work few hours, an aspect we could not consider in this study. More research is needed to better understand the role of age in this association.

### Strengths and limitations

To our knowledge, this is the first study investigating the association between workplace sexual and gender harassment and psychotropic medication. Major strengths of the study are the large and fairly representative sample of the Swedish working population with very limited missing data, exposure measurements including harassment frequency and differentiating between type of harasser, the long follow-up time and linkage to register data on the purchase of psychotropic medication, an objective measure of mental ill health that was assessed by medical professionals as grounds for pharmaceutical treatment. This being said, while the Swedish Prescribed Drug Register is highly reliable, it provides no information on specific diagnoses or patients’ adherence with the treatment, and psychotropics are not exclusively prescribed based on psychiatric diagnoses. Also, the determination of participants’ gender with register data does no justice to the complexities of gender identity, and some misclassification of transgender or non-binary participants can be assumed. Furthermore, potential selection bias must be acknowledged, as the response rate was lower in younger individuals and those with low education, income or of non-Swedish origin.^41^ This differential attrition may limit the generalisability of our results. Also, respondents had to self-label the exposures as sexual harassment or respectively harassment based on their gender. As respondents are consistently found to acknowledge only rather severe experiences that constitute a small fraction of what researchers classify as harassment cases,^17 42^ this measure can be assumed to have led to underreporting and in consequence an underestimation of the associations due to non-differential misclassification. Also, the exposure measures were unspecific regarding the gender and status of the harasser (colleague or superior), factors that are known to be relevant for the impact of the mistreatment. Furthermore, in some exposure groups, low statistical power limited the possibility to detect associations. On the other hand, residual confounding cannot be ruled out. Individuals with a history of mental ill health might more often work in employment situations where GBVH is more prevalent; they might also get more targeted or be more inclined to acknowledge harassing behaviour, for example, due to prior experiences with GBVH. While the exclusion of individuals who purchased psychotropic medication at baseline reduces these potential biases, it could also be an overcontrol in some cases. We could not determine the onset of the GBVH, and some of the excluded respondents might have experienced the GBVH under a longer time, and therefore, received pharmaceutical treatment in the year of survey participation. Taken together, this study was based on sound methodology and is the first to suggest an elevated risk of prospective treatment with psychotropic medication in workers experiencing sexual or gender harassment.

## Conclusion and implications

Our results highlight the importance of recognising the harm caused by gender harassment in the workplace. The construct captures a spectrum of experiences from blatant to more covert expressions of disrespect for individuals due to their gender, all of which organisations need to pay more attention to. Also, healthcare professionals would be well advised to consider sexual and gender harassment as potential factors in the aetiology of mental ill health.

## Data Availability

Data may be obtained from a third party and are not publicly available. Due to legal restrictions, the data are not open to the public. To ensure the integrity and privacy of the study participants, publishing the data set underlying the study is not permitted. Data requests may be addressed to the SLOSH data manager, Constanze Leineweber at Constanze.leineweber@su.se.
