# Supplementary material for: Sexual and gender harassment and use of psychotropic medication among Swedish workers: a prospective cohort study
Source: Occup Environ Med. 2022 Mar 9;79(8):507–13. doi: 10.1136/oemed-2021-108087 (PMC9304105; doi:10.1136/oemed-2021-108087)
Supplement: Supplementary data [file oemed-2021-108087supp001.pdf]

APPENDIX

Analyses stratified by gender

Table A1 Results from Cox regression analyses on the association between different combinations of work-related sexual and gender harassment (GBVH) and psychotropics for women and men separately, presented as hazard ratios (HR) and 95% confidence intervals.

	Women			Men		
	Exposed	Psychotropics		Exposed	Psychotropics	
	N (%)	N	HR (95% CI)	N (%)	N	HR (95% CI)
**GBVH**						
Not in 12 months	9 511 (83.2)	2 107	1	10 417 (94.4)	1 430	1
Gender harassment/no sexual harassment	982 (9.0)	242	1.16 (1.01 to 1.33)	370 (3.4)	65	1.30 (1.01 to 1.67)
Sexual harassment/no gender harassment	612 (5.4)	164	1.17 (1.00 to 1.38)	157 (1.4)	29	1.25 (0.86 to 1.81)
Sexual and gender harassment	331 (2.9)	90	1.27 (1.03 to 1.57)	87 (0.8)	16	1.30 (0.79 to 2.14)

Adjusted for survey year, age, education, family situation, income, parental migration background, managerial responsibilities, and industry classification.

Table A2 Results from Cox regression analyses on the association between work-related sexual harassment (SH) and gender harassment and psychotropics for women and men separately, presented as hazard ratios (HR) and 95% confidence intervals.

	Women			Men		
	Exposed	Psychotropics		Exposed	Psychotropics	
	N	N	HR (95% CI)	N	N	HR (95% CI)
**Workplace SH by any harasser**						
Not in 12 months	10 493	2 349	1	11 787	1 495	1
Once in 12 months	658	172	1.13 (0.96 to 1.32)	140	24	1.14 (0.76 to 1.71)
Monthly to daily	224	68	1.33 (1.06 to 1.66)	73	14	1.39 (0.90 to 2.14)
**Gender harassment**						
Not in 12 months	10 123	2 271	1	10 574	1 459	1
Once in 12 months	881	225	1.19 (1.04 to 1.37)	324	59	1.31 (1.01 to 1.70)
Monthly to daily	432	107	1.14 (0.93 to 1.38)	133	22	1.23 (0.81 to 1.88)

Adjusted for survey year, age, education, family situation, income, parental migration background, managerial responsibilities, and industry classification.

Analyses stratified by age

Table A3 Results from Cox regression analyses on the association between different combinations of work-related sexual and gender harassment (GBVH) and psychotropics for age groups separately, presented as hazard ratios (HR) and 95% confidence intervals.

	16-25<br>HR (95% CI)	26-35<br>HR (95% CI)	36-45<br>HR (95% CI)	46-55<br>HR (95% CI)	56-64<br>HR (95% CI)
N	1 858	4 032	5 868	5 969	4 740
**GBVH**					
Not in 12 months	1	1	1	1	1
Gender harassment/no sexual harassment	0.92 (0.55 to 1.55)	1.35 (1.06 to 1.73)	1.18 (0.94 to 1.47)	1.16 (0.91 to 1.47)	1.18 (0.88 to 1.58)
Sexual harassment/no gender harassment	1.31 (0.91 to 1.87)	1.12 (0.83 to 1.52)	1.08 (0.77 to 1.50)	1.23 (0.92 to 1.65)	1.26 (0.80 to 2.00)
Sexual and gender harassment	0.80 (0.43 to 1.48)	1.37 (0.97 to 1.93)	1.39 (0.95 to 2.05)	1.17 (0.73 to 1.87)	1.72 (1.00 to 3.00)

Adjusted for survey year, gender, education, family situation, income, parental migration background, managerial responsibilities, and industry classification.

Table A4 Results from Cox regression analyses on the association between work-related sexual harassment (SH) and gender harassment and psychotropics for age groups, presented as hazard ratios (HR) and 95% confidence intervals.

	16-25<br>HR (95% CI)	26-35<br>HR (95% CI)	36-45<br>HR (95% CI)	46-55<br>HR (95% CI)	56-64<br>HR (95% CI)
**N**	1 858	4 032	5 868	5 969	4 740
**Workplace SH by any harasser**					
Not in 12 months	1	1	1	1	1
Once in 12 months	1.01 (0.67 to 1.52)	1.07 (0.81 to 1.42)	1.06 (0.78 to 1.46)	1.20 (0.90 to 1.61)	1.47 (0.97 to 2.21)
Monthly to daily	1.31 (0.83 to 2.07)	1.34 (0.93 to 1.92)	1.42 (0.95 to 2.14)	1.18 (0.74 to 1.89)	1.32 (0.68 to 2.57)
**Gender harassment**					
Not in 12 months	1	1	1	1	1
Once in 12 months	0.70 (0.42 to 1.17)	1.39 (1.09 to 1.77)	1.28 (1.02 to 1.60)	1.19 (0.92 to 1.53)	1.23 (0.90 to 1.67)
Monthly to daily	1.15 (0.63 to 2.12)	1.22 (0.87 to 1.72)	1.08 (0.75 to 1.54)	1.12 (0.78 to 1.62)	1.27 (0.81 to 1.99)

Adjusted for survey year, gender, education, family situation, income, parental migration background, managerial responsibilities, and industry classification.

Sensitivity analyses with 2-year follow-up

Table A5 Sensitivity analysis: Results from Cox regression analyses on the association between different combinations of work-related sexual and gender harassment (GBVH) and psychotropics, follow-up censored to 2 years, presented as hazard ratios (HR) and 95% confidence intervals.

	Exposed	Psychotropics	
	N	N	HR (95% CI)
**GBVH**			
Not in 12 months	20 428	2 193	1
Gender harassment/no sexual harassment	1 381	190	1.20 (0.98 to 1.45)
Sexual harassment/no gender harassment	789	130	1.08 (0.83 to 1.40)
Sexual and gender harassment	426	61	1.45 (1.08 to 1.96)

Adjusted for survey year, gender, age, education, family situation, income, parental migration background, managerial responsibilities, and industry classification.

Table A6 Sensitivity analysis: Results from Cox regression analyses on the association between work-related sexual harassment (SH) and gender harassment and psychotropics, follow-up censored to 2 years, presented as hazard ratios (HR) and 95% confidence intervals.

	Exposed	Psychotropics	
	N	N	HR (95% CI)
**Workplace SH by any harasser**			
Not in 12 months	21 280	2 383	1
Once in 12 months	798	131	1.04 (0.81 to 1.34)
Monthly to daily	389	60	1.51 (1.11 to 2.04)
**SH by superiors or colleagues**			
Not in 12 months	22 125	2 522	1
Once in 12 months	207	34	1.10 (0.35 to 3.43)
Monthly to daily	135	18	1.66 (0.62 to 4.45)
**SH by others (e.g., clients)**			
Not in 12 months	21 512	2 419	1
Once in 12 months	658	107	1.43 (0.71 to 2.88)
Monthly to daily	297	48	1.29 (0.54 to 3.13)
**Gender harassment**			
Not in 12 months	20 697	2 323	1
Once in 12 months	1 205	170	1.47 (0.99 to 2.20)
Monthly to daily	565	81	1.58 (0.87 to 2.87)

Adjusted for survey year, gender, age, education, family situation, income, parental migration background, managerial responsibilities, and industry classification.

Sensitivity analyses with full study sample (no exclusion due to psychotropics use), adjusted for previous medication

Table A7 Sensitivity analysis: Analyses with the full study sample, not excluding individuals who had purchased medication in the survey year. Analyses adjusted for previous psychotropics use.

	Exposed	Psychotropics	
	N	N	HR (95% CI)
**GBVH**			
Not in 12 months	22 280	3 537	1
Gender harassment/no sexual harassment	1 579	307	1.14 (1.02 to 1.29)
Sexual harassment/no gender harassment	868	193	1.22 (1.05 to 1.42)
Sexual and gender harassment	485	106	1.27 (1.05 to 1.55)

Adjusted for survey year, gender, age, education, family situation, income, parental migration background, managerial responsibilities, industry classification, previous psychotropics.

Table A8 Sensitivity analysis: Analyses with the full study sample, not excluding individuals who had purchased medication in the survey year. Analyses adjusted for previous psychotropics use.

	Exposed	Psychotropics	
	N	N	HR (95% CI)
**Workplace SH by any harasser**			
Not in 12 months	23 859	3 844	1
Once in 12 months	909	196	1.16 (1.00 to 1.34)
Monthly to daily	444	103	1.34 (1.10 to 1.63)
**SH by superiors or colleagues**			
Not in 12 months	24 810	4 061	1
Once in 12 months	256	48	1.02 (0.76 to 1.35)
Monthly to daily	146	34	1.45 (1.03 to 2.03)
**SH by others (e.g., clients)**			
Not in 12 months	24 131	3 898	1
Once in 12 months	739	163	1.18 (1.00 to 1.38)
Monthly to daily	342	82	1.35 (1.09 to 1.68)
**Gender harassment**			
Not in 12 months	23 148	3 730	1
Once in 12 months	1 393	284	1.19 (1.06 to 1.35)
Monthly to daily	671	129	1.09 (0.92 to 1.30)

Adjusted for survey year, gender, age, education, family situation, income, parental migration background, managerial responsibilities, industry classification, and previous psychotropics.
